# O8.3. CLINICAL AND FUNCTIONAL OUTCOMES IN YOUNG ADULTHOOD OF CHILDREN WITH PSYCHOTIC SYMPTOMS: A LONGITUDINAL TWIN COHORT STUDY

**DOI:** 10.1093/schbul/sby015.239

**Published:** 2018-04-01

**Authors:** Antonella Trotta, Louise Arseneault, Avshalom Caspi, Andrea Danese, Terrie Moffitt, Carmine Pariante, Helen Fisher

**Affiliations:** 1 Institute of Psychiatry, Psychology & Neuroscience, King’s College London; 2 King’s College London; 3 King’s College London, Duke University

## Abstract

**Background:**

Childhood psychotic symptoms, such as hallucinations and delusions, are relatively common and have been shown to increase risk of psychotic disorders in adulthood. However, less is known about their association with other forms of psychopathology and more broadly with social and occupational functioning during the crucial transition to adulthood. Using a prospective genetically-sensitive birth cohort we investigated associations between age-12 psychotic symptoms and a range of mental health problems and functional outcomes at age 18.

**Methods:**

Data from utilized from the Environmental Risk (E-Risk) Longitudinal Twin Study, a birth cohort of 2,232 twins born in 1994–1995 in England and Wales, followed to age 18 with 93% retention. Childhood psychotic symptoms were assessed in private interviews at age 12. At age 18, interviews were conducted to assess psychopathology, social and occupational functioning, physical health, quality of life, risky and offending behaviors.

**Results:**

Children with psychotic symptoms were at greater risk of psychotic phenomena, depression, anxiety, and suicide attempts or self-harm in young adulthood than children without such symptoms. They were also more likely to be obese, smoke cigarettes, be lonely, already have children, and report a lower quality of life at age 18 compared with their unaffected peers. These associations held when controlling for sex, age-5 IQ, other psychopathology at age 12, and family environment.

**Discussion:**

In our genetically sensitive cohort, we showed strong evidence of continuity between early psychotic symptoms in childhood and persistence of psychotic phenomena to young adulthood. Psychotic symptoms in childhood are also important risk markers for a wide range of non-psychotic disorders and poor functional outcomes and therefore should be carefully assessed and treated to prevent adverse consequences in adulthood.

